# CSE/H_2_S system alleviates uremic accelerated atherosclerosis by regulating TGF-β/Smad3 pathway in 5/6 nephrectomy ApoE^−/−^ mice

**DOI:** 10.1186/s12882-020-02183-z

**Published:** 2020-12-04

**Authors:** Xiangxue Lu, Shixiang Wang, Sujuan Feng, Han Li

**Affiliations:** Department of Blood Purification, Beijing Chao-Yang Hospital, Capital Medical University, No. 8 Gongti South Road, Chaoyang District, Beijing, 100020 China

**Keywords:** Uremia accelerated atherosclerosis, ApoE^−/−^ mice, Hydrogen sulfide, TGF-β/Smad pathway, 5/6 nephrectomy

## Abstract

**Background:**

Hydrogen sulfide (H_2_S) has been shown to inhibit the atherosclerosis development and progression. It is produced by cystathionine γ-lyase (CSE) in the cardiovascular system. In our previous study, it has been shown that CSE/H_2_S system plays a significant role in the changes of uremic accelerated atherosclerosis (UAAS), but the mechanism is not known clearly.

**Methods:**

In this study, we explored the antagonism of CSE/H_2_S system in UAAS and identified its possible signaling molecules in ApoE^−/−^ mice with 5/6 nephrectomy and fed with atherogenic diet. Mice were divided into sham operation group (sham group), UAAS group, sodium hydrosulfide group (UAAS+NaHS group) and propargylglycine group (UAAS+PPG group). Serum creatinine, urea nitrogen, lipid levels and lesion size of atherosclerotic plaque in the aortic roots were analyzed. Meanwhile, the expression of CSE, TGF-β and phosphorylation of Smad3 were detected.

**Results:**

Compared with sham group, the aortic root of ApoE^−/−^ mice in the UAAS group developed early atherosclerosis, the levels of total cholesterol, triglyceride, low-density lipoprotein-cholesterol, serum creatinine and urea nitrogen were also higher than that in the sham group. NaHS administration can inhibit the development of atherosclerosis, but PPG administration can accelerate the atherosclerosis development. Meanwhile, the protein expression levels of CSE and TGF-β and phosphorylation of Smad3 significantly decreased in the UAAS mice. Treatment of UAAS mice with NaHS inhibited TGF-β protein expression and Smad3 phosphorylation decrease, but PPG treatment had the opposite effect.

**Conclusions:**

The CSE/H_2_S system is of great importance for treating atherosclerosis in patients with chronic kidney disease, and it may protect the vascular from atherosclerosis through the TGF-β/Smad pathway.

## Background

Extensive atherosclerosis is a well-documented complication of advanced chronic kidney disease (CKD), which is related to a high incidence of cardiovascular events and mortality [1], the contributing factors include inflammation, oxidative stress and blood vascular endothelial cells dysfunction. The incidence age of atherosclerosis in end-stage renal disease patients is advanced, and the disease progression is faster, which is known as uremia accelerated atherosclerosis (UAAS) [2]. Atherosclerosis is often caused by a complex interplay between environmental factors and genetic. The high expression level of a series of chemokines and adhesion molecules in the endothelium could attract monocytes to the intima of the artery, then the monocytes may become foam cells through taking up lipids [3]. Meanwhile, the growing evidence showed that endothelial dysfunction is the key role in the atherosclerotic plaque development and progression and many kinds of atherosclerotic complications occurrence [4]. There are many non-traditional risk factors for atherosclerosis in patients with CKD, such as hyperhomocysteinemia, increased oxidative stress and anemia [5].

Transforming growth factor-beta (TGF-β) is the prototypical member of secreted growth factors [6]. Disturbed TGF-β signaling can cause many kinds of disorders such as atherosclerosis and cardiac fibrosis [7]. There is still a debate about whether TGF-β plays an anti- or a pro- atherogenic role in the atherosclerosis development. Mallat Z et al. confirmed that inhibition of TGF-β signaling could accelerate the development of atherosclerosis with decreased collagen content and increased inflammatory component in CSE^−/−^ mice [8]. Volger et al. demonstrated that activation of the TGF-β signaling pathway intermediate Smad3 in endothelium atherosclerotic plaques [9]. Conversely, Lutgens et al. demonstrated that inhibition TGF-β signaling by using a recombinant soluble TGF-β receptor II could decrease the area of atherosclerotic plaque and alter the balance between fibrosis and plaque inflammation in ApoE^−/−^ mice [10]. Similarly, there is evidence that TGF-β played an anti-atherosclerosis role for its partial disruption of vascular cell adhesion molecule 1 (VCAM-1), and intercellular adhesion molecule 1 (ICAM-1) expression in mouse models of accelerated atherosclerosis [11].

Hydrogen sulfide (H_2_S) is an important signaling molecule with many kinds of physiological functions as the third gaseous medium following carbon monoxide and nitric oxide. In mammalian tissues, H_2_S is endogenously produced mainly by cystathionine γ-lyase (CSE) and cystathionine β-synthase (CBS) from L-cysteine, homocysteine and cystathione, and is also produced by the catalysis of 3-mercaptopyruvate sulfurtransferase (3-MST) from L-cysteine and/or homocysteine [12]. Emerging data demonstrated that CSE/H_2_S system plays an essential role in the cardiovascular systems [13]. In our previous study, we have confirmed that plasma H_2_S was negative correlated with cardiovascular disease and mortality in chronic hemodialysis patients [14]. Furthermore, abnormalities of H_2_S and TGF-β/Smad signaling pathway could lead to UAAS development in chronic hemodialysis patients with diabetic nephropathy [15]. We also found that endogenous CSE/H_2_S system may protect against the formation of UAAS via cPKCβII/Akt signal pathway [16]. But how the 5/6 nephrectomy and CSE/H_2_S system affect the plaque formation and whether other signaling pathways may also participate in this process were still unclear in the previous study. Therefore, we determined the effects of CSE/H_2_S system and TGF-β/Smad signaling pathway on the development of atherosclerosis in UAAS mice, and identified the possible molecular mechanisms in this study.

## Methods

### Animals and animal models

All procedures were approved by the experimental animal ethics committee of Beijing Chao-Yang Hospital, Capital Medical University (Permit Number: 2016-KE-12). The Guide for the Care and Use of Laboratory Animals of the National Institutes of Health was followed in this study. ApoE^−/−^ mice used in the study were 6–8 weeks old and weighed 20–25 g. Male ApoE^−/−^ mice were purchased from Beijing Vital River Laboratory Animal Technology Co., Ltd. (License No. SCXK (Beijing) 2016–0006). Animals were housed in a room under a 12:12-h light/dark cycle with free access to food and water in metal breeding cages. The humidity was maintained at 55% and the temperature was maintained at 23 °C. High-fat diet was purchased from Research Diets (Cat. No.: D12108C).

ApoE^−/−^ mice were divided into four groups randomly depending on treatment (*n* = 6 per group) [17]. The mice in the sham group were performed sham surgery with decapsulation of both kidneys and fed with a high-fat diet; UAAS group mice were fed with a high-fat diet and subjected to 5/6 nephrectomy; the UAAS group mice intraperitoneal injected with NaHS (H_2_S donor, 56 μmol/kg body weight/day) were considered as the UAAS+NaHS group; the UAAS group mice intraperitoneal injected with PPG (CSE inhibitor, 37.5 mg/kg body weight/day) were considered to the UAAS+PPG group. NaHS and PPG were dissolved in normal saline and NaHS was freshly prepared each day.

The 5/6 nephrectomy was performed under isoflurane anesthesia in two steps as previously described [18]. The mice were anesthetized in an anesthesia chamber filled with 1.5% isoflurane in a mixture of O_2_ and N_2_ (50%/50%) at a flow rate of 0.9–1 L/min during the surgery. In short, at the first stage, a left flank incision was made, the left renal capsule was removed to avoid damage to the ureter and adrenal, then the upper and lower poles were partially resected. Two weeks later, a right flank incision was made and the right nephrectomy was performed. In the sham group, a left flank incision was performed at the first stage, then the flank incision was closed after the upper and lower poles of kidney were identified. Two weeks later, a right flank incision was performed, then the flank incision was closed after the right renal artery was identified. Mice were euthanized via cervical dislocation according to the American Veterinary Medical Association.

### Measurement of serum creatinine, urea nitrogen and lipids profile

Mice were fasted for at least 8 h before the blood collection, and blood was collected into anticoagulant tubes. The levels of serum creatinine (Scr), urea nitrogen (BUN), total cholesterol (TC), triglyceride (TG), and low-density lipoprotein-cholesterol (LDL-C) were determined by a Hitachi 7180 Autoanalyzer (Hitachi, Tokyo, Japan) at 2, 4, 6, 8, and 10 weeks after nephrectomy.

### Assessment of aortic atherosclerotic lesions

To detect the atherosclerotic lesions at 2, 4, 6, 8, and 10 weeks after nephrectomy, 8–10 sections taken from every four consecutive sections of aortic arch were stained with hematoxylin-eosin [19] and Oil Red O [20] method. Images were collected on an Olympus FSX100 microscope (Olympus, Tokyo, Japan). The atherosclerotic lesion areas were quantified using Image J software.

### Measurement of H_2_S concentration in the plasma

The blood was collected in EDTA-coated tube, after centrifugation at 500 g for 5 min, the plasma was immediately obtained and rapidly added to the assay mixture. The measurement method of plasma H_2_S concentration was described previously [14]. In brief, 100 μL of plasma was added into a test tube containing 2.5 mL of distilled water and 0.5 mL of 1% zinc acetate, then N, N-dimethyl-p-phenylenediamine dihydrochloride (20 mM, 500 μL) in 7.2 M HCl and FeCl3 (30 mM, 400 μL) in 1.2 M HCl were added. This mixture was incubated at room temperature for 20 min, then 1 mL of 10% trichloroacetic acid was added to the solution and centrifuged at 4000×g for 10 min to remove the protein in the plasma. The optical absorbance of the supernatant at 670 nm was measured with a spectrometer. All samples were assayed repeatedly, and the H_2_S concentration in the solution was calculated against the calibration curve of the standard NaHS solution.

### Western blot analysis

Total protein in aortic tissues were washed twice with ice-cold PBS and solubilized in buffer A [5 mM Tris-Cl, pH 7.5, containing 2 mM dithiothreitol (DTT), 2 mM EDTA, 1 mM EGTA, 5 lg/mL each of leupeptin, aprotinin, pepstatin A and chymostatin, 50 mM potassium fluoride, 50 nM okadaic acid, 5 mM sodium pyrophosphate, and 2% sodium dodecyl sulfate (SDS)] and sonicated to dissolve the tissue completely. BCA kit (Pierce Company, Rockford, IL, USA) was used to determine the protein concentration. Proteins (40 μg) from each sample were loaded on 10% SDS-polyacrylamide gel electrophoresis. The gels were electrophoresed, and then transferred onto polyvinylidene difluoride (PVDF) membrane (GE Healthcare) at 4 °C. After rinsed with TTBS (20 mM Tris-Cl, pH 7.5, 0.15 M NaCl and 0.05% Tween-20), the transferred PVDF membrane was blocked with 10% skim milk in TTBS for 1 h and incubated with the CSE, TGF-β, p-Smad3 and total-Smad3 (Santa Cruz Biotechnology, Inc., CA 95060, USA, 1:1000 dilution) for 3 h. The membrane was washed and incubated with IRDye® 800CW Goat anti-Rabbit IgG or Goat anti-Mouse IgG secondary antibody (1:5000 dilution) at room temperature for 2 h followed by LiCor Odyssey gel imaging scanner detection (LI-COR Biosciences, Lincoln, NE). To verify equal loading of protein, the blots were reprobed with primary monoclonal antibody against β-actin (ProteinTech Company, USA).

### Immunohistochemistry for CSE in the aortic root

In brief, 6-μm cross-section slides were incubated with anti-CSE primary antibodies at 4 °C overnight. After incubated with appropriate biotinylated secondary antibodies and HRP streptavidin, diaminobenzidine (DAB) was added for color development and hematoxylin for counterstaining. Images were collected on an Olympus FSX100 microscope (Olympus, Tokyo, Japan) and quantified using ImagePro plus 6.0 software.

### Statistical analysis

The statistical software package (SPSS for Window, Version 21.0, SPSS, USA) was used to analyzed the data. Measurement data was presented as mean value ± standard deviation (±SD). Comparisons among groups were performed using one-way ANOVA followed by the post-hoc Dunnett test, and comparisons between two groups were performed using the two-sample t-test. Quantitative-One software (Gel Doc 2000 imaging system, Bio-Rad Company, CA, USA) was used for quantitative analysis of immunoblotting results. A *P* value < 0.05 was considered statistically significant.

## Results

### The effect of CSE/H_2_S system on the levels of Scr and lipids in UAAS mice

**Fig. 1:**
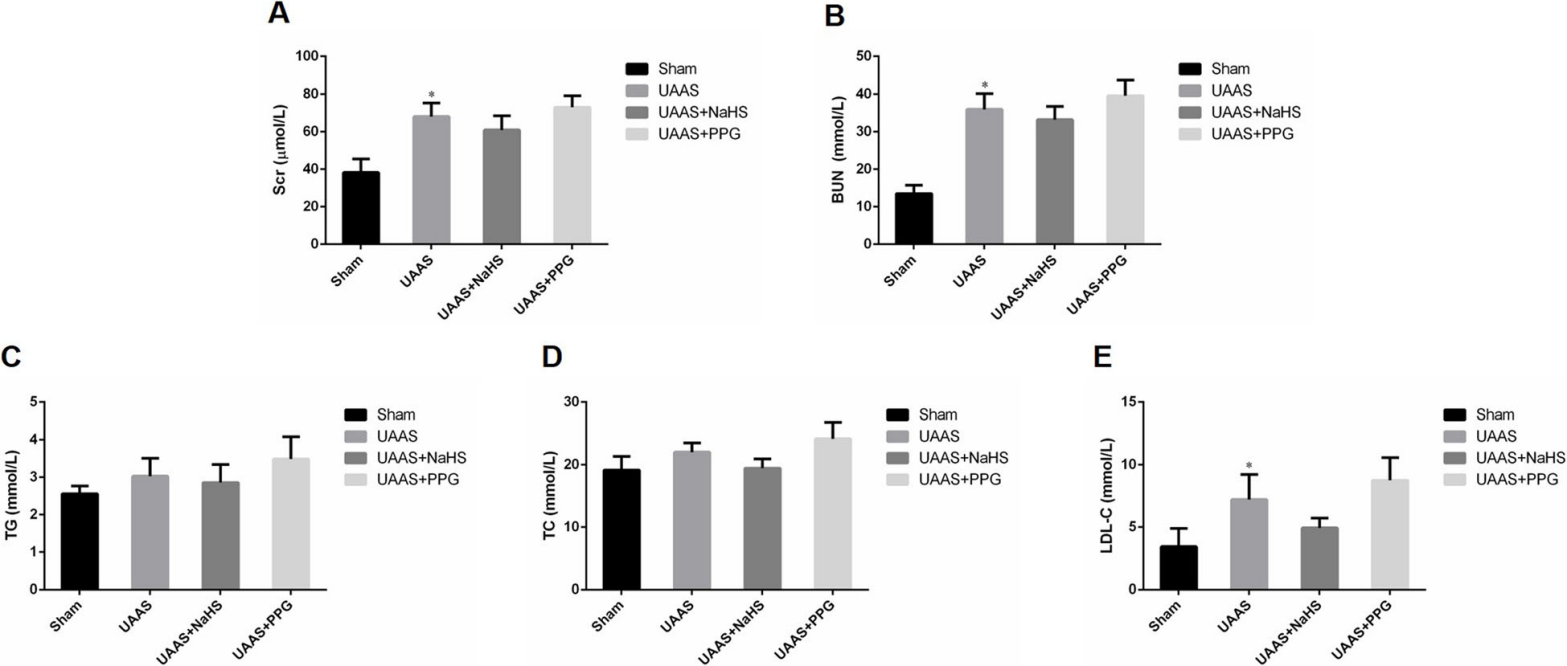
Changes in Scr, BUN and levels of lipids in ApoE^−/−^ mice at 10 weeks after operation. Compared with sham group, UAAS mice showed significantly increased level of Scr and BUN; Scr and BUN were increased in UAAS+PPG group mice compared with UAAS group mice (**a** and **b**). Meanwhile, LDL-C was significantly increased in UAAS group compared with sham group at 10 weeks after operation (**e**) (*P < 0.05 vs. sham group, *n* = 6 per group)

The levels of Scr, BUN and lipids were measured in all mice at 2, 4, 6, 8, and 10 weeks after operation and were shown in Supplementary Figure 1. Scr and BUN and levels of lipids were increased in UAAS, UAAS+NaHS and UAAS+PPG group mice when compared with sham group mice. Statistical analysis of these values at 10 weeks after operation showed that compared with sham group, UAAS mice showed significantly increased level of Scr and BUN (Fig. 1a and b). Meanwhile, LDL-C was significantly increased in UAAS group compared with sham group at 10 weeks after operation (Fig. 1e).

### Effect of CSE/H_2_S system on the size of aortic atherosclerotic plaque

**Fig. 2:**
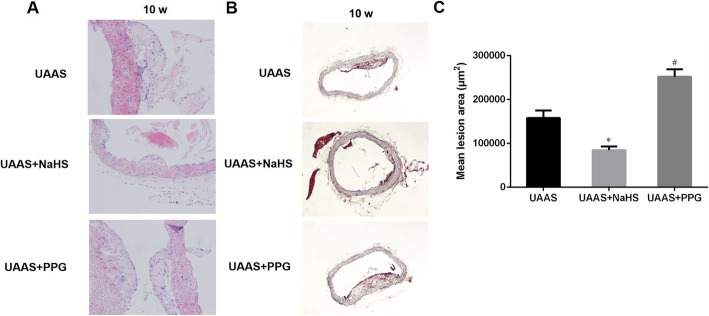
Effect of CSE/H_2_S system on aortic root lesion in UAAS mice at 10 weeks after operation. HE stained (**a**) and Oil Red O stained (**b**) aortic root lesions of mice at 10 weeks after operation (40×) showed the size of atherosclerotic plaque in the aortic root. Statistical analysis of the aortic root lesions in UAAS mice at 10 weeks after operation (**c**) showed that compared with UAAS group, the aortic root lesions were significantly alleviated in UAAS+NaHS mice, but deteriorated in UAAS+PPG mice at 10 weeks after operation (*P < 0.05 vs. UAAS group, and ^#^*P* < 0.05 vs. UAAS group, *n* = 6 per group)

To confirm the effect of CSE/H_2_S system on the aorta, HE staining was used to detect the of aortic atherosclerotic plaque formation. The result in supplementary figure 2 showed that the aortic atherosclerotic plaque was widely observed in UAAS group mice after 6 weeks operation. Compared with the UAAS group, the size of plaque was significantly diminished in UAAS+NaHS group mice between 6 to 10 weeks after operation, but the atherosclerotic plaque had been observed after 4 weeks operation in UAAS+PPG group mice. Statistical analysis of the lesion areas at 10 weeks after operation showed that the aortic root lesions were significantly alleviated in UAAS+NaHS mice, but deteriorated in UAAS+PPG mice (Fig. 2).

### Changes in CSE/H_2_S system in UAAS mice

**Fig. 3:**
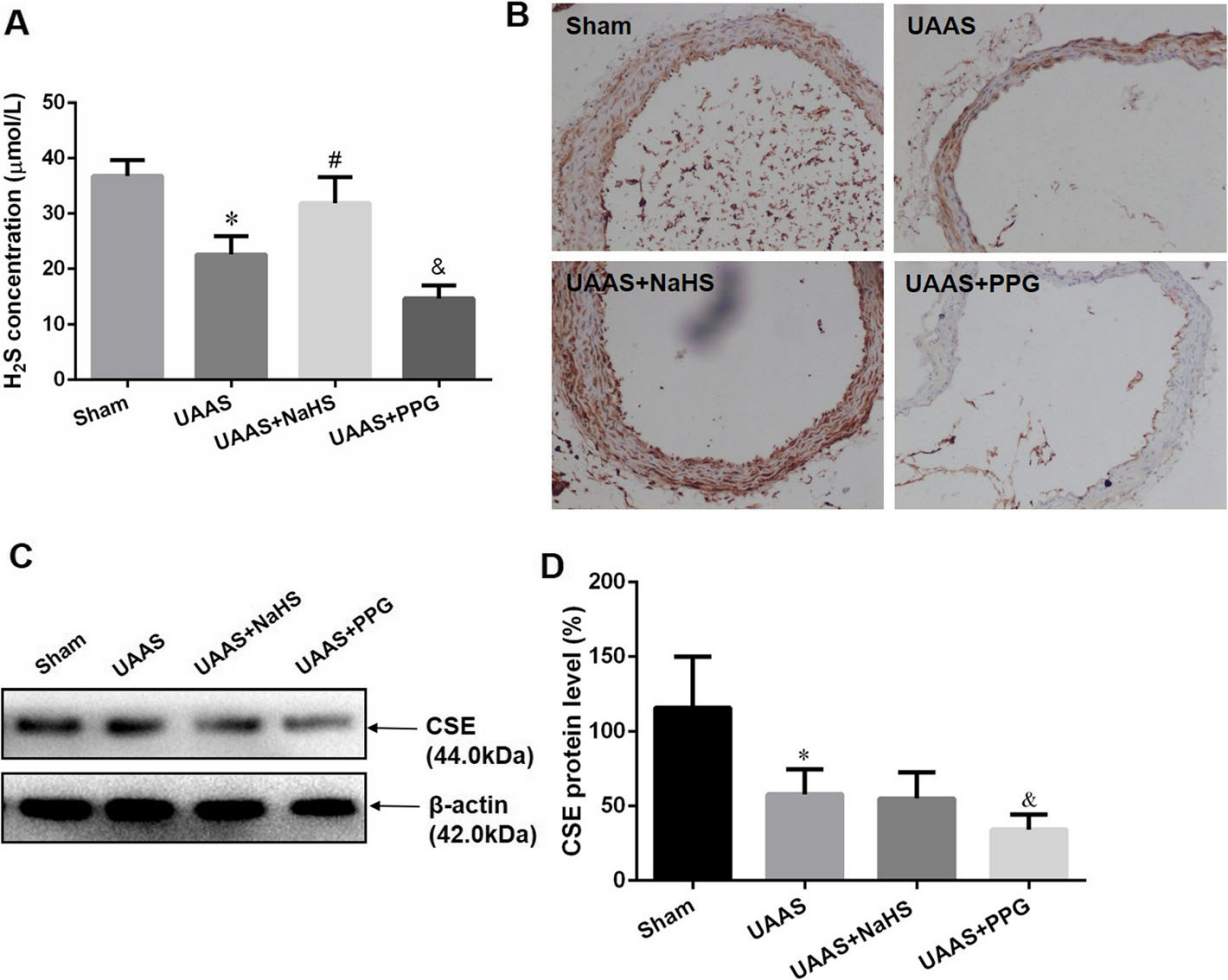
Changes in CSE/H_2_S system during UAAS. H_2_S concentration was determined by zinc acetate (**a**). Compared with sham mice, UAAS mice showed significantly lower H_2_S level in plasma, NaHS treatment alleviated the decrease of H_2_S level, but PPG administration aggravated the decrease of H_2_S level in UAAS mice (*P < 0.05 vs. sham group, ^#^*P* < 0.05 vs. UAAS group, and ^&^*P* < 0.05 vs. UAAS group, *n* = 6 per group). CSE protein expression was detected by immunohistochemistry (**b**) and Western blot (**c**, **d**) respectively. Compared with sham mice, the CSE protein expression in aorta of UAAS mice significantly decreased, NaHS treatment did not influence the protein expression, but intraperitoneal injected PPG resulted in the expression of CSE lower than that in UAAS group (*P < 0.05 vs. sham group, and ^&^*P* < 0.05 vs. UAAS group, *n* = 6 per group)

6 weeks after operation, UAAS mice showed significantly lower H_2_S level in plasma when compared with sham mice. When treated with NaHS in UAAS mice, plasma H_2_S was significantly increased, and PPG treatment resulted in were plasma H_2_S significantly decreased compared to UAAS mice (Fig. 3a). Meanwhile, the immunohistochemistry and Western blot for CSE showed that NaHS treatment did not influence the protein expression of CSE in aorta of UAAS mice, but intraperitoneal injection of PPG resulted in the expression of CSE lower than that in UAAS group (Fig. 3b, c and d).

### Effect of CSE/H_2_S system on TGF-β/Smad3 pathway in UAAS mice

**Fig. 4:**
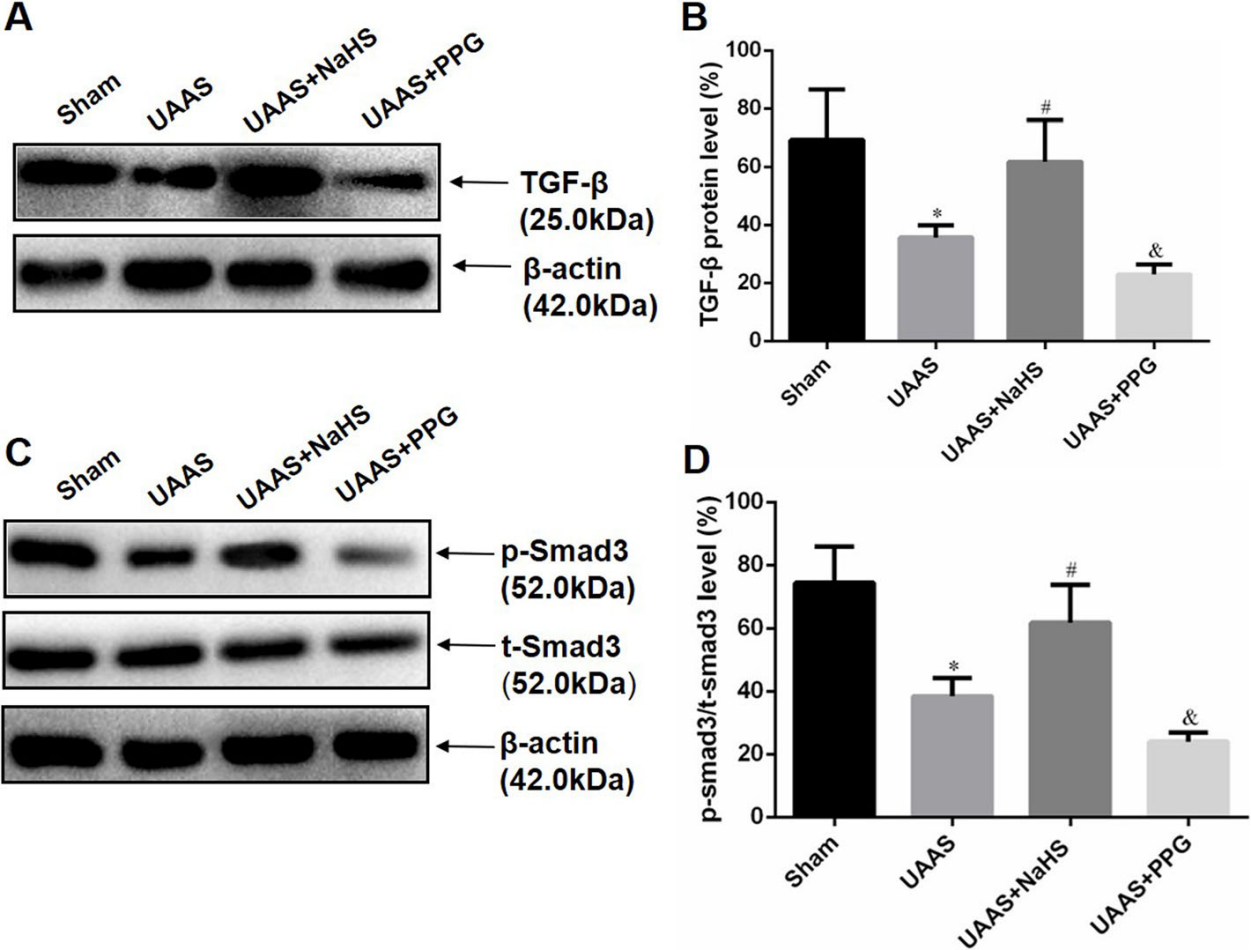
Effect of CSE/H_2_S system on TGF-β protein expression and Smad3 phosphorylation. Representative results of Western blot demonstrated the changes in TGF-β protein expression and Smad3 phosphorylation levels in the sham, UAAS, UAAS+NaHS and UAAS+PPG groups (**a**, **c**); Quantitative analysis (**b**, **d**) showed that TGF-β protein expression and Smad3 phosphorylation levels decreased significantly in UAAS mice, but NaHS treatment attenuated the UAAS-induced inhibition on TGF-β protein expression and Smad3 phosphorylation levels, and PPG treatment aggravated the decrease of TGF-β protein expression and Smad3 phosphorylation levels (*P < 0.05 vs. sham group, ^#^*P* < 0.05 vs. UAAS group, and ^&^*P* < 0.05 vs. UAAS group, *n* = 6 per group)

To explore the protective mechanism of CSE/H_2_S system in UAAS mice, we further detected the change of protein expression level of TGF-β and phosphorylation of Smad3 when CSE/H_2_S system was interfered. Figure 4 showed that the TGF-β protein expression and Smad3 phosphorylation in UAAS mice decreased when compared with sham group, and NaHS injection could suppress the degradation of both TGF-β and Smad3 phosphorylation, but PPG administration resulted in more decrease of TGF-β protein level and Smad3 phosphorylation.

## Discussion

ApoE^−/−^ mouse tend to develop atherosclerotic lesions spontaneously on a standard chow diet and are the most popular and convenient mouse model used to investigate atherosclerosis currently [21]. As shown in the previous studies [1, 22], chronic kidney disease is associated with profound vascular remodeling, which is characterized by intimal hyperplasia, accelerated atherosclerosis, excessive vascular calcification, and vascular stiffness. ApoE^−/−^ mice were excellent model for experimental atherosclerosis research [23]. It was shown that uremia accelerates both atherosclerosis and arterial calcification in ApoE^−/−^ mice after 5/6 nephrectomy [24]. The accumulation of uremic toxins in chronic kidney disease induces inflammation, oxidative stress and endothelial dysfunction, which is a key step in atherosclerosis [25]. Unlike previous studies mainly focused on vascular calcification, the present study focused on atherosclerosis related to endothelial dysfunction, which was not widely studied and the mechanisms of which were not well understood and considered to be an innovation of this research. Meanwhile, several articles [26–28] have explored the effect of H_2_S on atherosclerosis, but none of them were associated with uremia. This study is the first to use ApoE^−/−^ mice to characterize the role of endogenous CSE/H_2_S system in UAAS progression. The results obtained showed the elevated levels of Scr, BUN, TC, TG and LDL-C, which demonstrated that 5/6 nephrectomy can induce the renal failure and fed mice with a high-fat diet can further accelerate the atherosclerotic progression in mice with uremia. The plasma H_2_S level and aortic CSE expression were significantly decreased when ApoE^−/−^ mice subjected to UAAS development, which indicated that the downregulation of CSE/H_2_S system probably contribute to atherosclerotic progression.

NaHS can be completely metabolized in a short time and is known as a short-lived H_2_S donor [29]. In solution, NaHS can dissociates into Na^+^ and HS^−^, then the HS^−^ associates with H^+^ to produce H_2_S, compared with H_2_S gas, NaHS enables definition of the H_2_S concentrations in solution more accurate and reproducible, So the NaHS was used as a H_2_S donor [28]. Meanwhile, PPG was used as an inhibitor of H_2_S production because it is a potent, active site-directed, irreversible inhibitor of CSE [30]. In our study, we used NaHS and PPG to explore the role of endogenous H_2_S in ApoE^−/−^ mice UAAS pathogenesis, and found that treatment of NaHS significantly elevated the H_2_S level in plasma in the aorta of ApoE^−/−^ mice but have no influence on the protein expression of CSE, whereas PPG reduced the plasma H_2_S level and also aorta CSE protein expression significantly.

CSE/H_2_S deficiency was confirmed to link to several cardiovascular diseases, such as atherosclerosis, hypertension and myocardial ischemia/reperfusion injury [31]. H_2_S was reported to suppresses atherosclerosis by inducing apoptosis, facilitating blood vessel relaxation and endothelium regeneration, suppressing proliferation of VSMCs [32], inhibiting reactive oxygen species production [33], adhesion molecule expression [28], foam cell formation [34] and anti-inflammation. Wang et al. found that the H_2_S exerted an antiatherogenic effect in ApoE^−/−^ mice for the first time, exogenous H_2_S supplementation increased the H_2_S level in plasma and reduced the size of atherosclerotic plaque in the aortic root of ApoE^−/−^ mice, whereas PPG reduced plasma H_2_S level and increased plaque size in the aorta [28]. Lin Y et al. confirmed that H_2_S could inhibit the proliferation and migration of VSMCs by upregulation of plasma NO and protein S-nitrosylation, thus inhibiting the development of atherosclerosis [35]. CSE^−/−^ mice were further used to detected the protective effect of H_2_S in atherosclerosis. ICAM-1 is a marker for vascular inflammation in atherosclerosis, which can be found significantly increased in atherosclerotic plaques. Mani S et al. reported that endogenously synthetic H_2_S can protect vascular tissues from atherogenic damage in CSE^−/−^ mice fed with a high cholesterol diet, which displayed an increase in aortic ICAM-1 mRNA and protein expression level and an increase of atherosclerotic plaque size compared to wild type control [36]. Yang et al. also observed that interaction between H_2_S and extracellular signal-regulated kinase (ERK)/MAPK played an important role in regulating VSMC proliferation and vascular remodeling in CSE^−/−^ mice [32]. However, the precise mechanism of CSE/H_2_S system on anti- atherosclerosis is still far from clear.

TGF-β family is mainly consisted of TGF-β1, β2 and β3 and more distantly related proteins like activins and inhibins, nodal proteins, and bone morphogenetic proteins (BMPs) [37]. TGF-βs are usually synthesized as an inactive precursor protein, which contains an amino-terminal signal peptide to direct it to the endoplasmatic reticulum. After removal of the signal peptide, the remaining propeptide is cleaved by the endoprotease furin to generate mature TGF-β [7]. TGF-βs regulate a large variety of cellular processes in many kinds of cell types, including the induction of proliferation, apoptosis, migration, adhesion, extracellular matrix protein production, inflammation and cytoskeletal organization [38]. Experiments based on human beings and mouse models supported that TGF-β/Smads signaling may participate in the development of atherosclerosis through modulated the fibrotic and inflammatory components of the vessel wall lesion. Panutsopulos D et al. indicated that high expression levels of TGF-β1 in human coronary artery correlated with advanced atherosclerosis [39]. Study from Chen PY et al. demonstrated that the fibroblast growth factor-dependent regulation of TGF-β activity played a significant role in the development of atherosclerotic lesions [40]. The anti-atherosclerotic effect of pioglitazone was probably mediated by the TGF-β/Smad signaling pathway [41]. However, some studies have shown an inverse relationship between TGF-β1 expression and atherosclerosis development. Bot PT et al. found that an increased expression of the TGFβ/Smad signaling pathway was helpful for stable plaque formation [42]. Frutkin AD et al. confirmed that TGF-β1 can limit atherosclerosis and prevent aortic dilation in ApoE^−/−^ mice [43]. Furthermore, increased expression of TGF-β1 in macrophage also reduced the development of atherosclerosis in ApoE^−/−^ mice [44].

The role of CSE/H_2_S system and TGF-β/Smad pathway in atherogenic process was discussed respectively in other studies. Meanwhile, we confirmed the protective effect of H_2_S regulating cPKCβII/Akt pathway in UAAS patients against atherosclerosis formation and cardiovascular disease progression in our previous study [16]. But whether other signaling pathways may also participate in this process and how the 5/6 nephrectomy and CSE/H_2_S system affect the plaque formation were still unclear in the previous study. In the present manuscript, we determined the effects of CSE/H_2_S system and TGF-β/Smad signaling pathway on the development of atherosclerosis in UAAS mice, and identified the possible molecular mechanisms in this study. It was found that UAAS mice showed significantly lower H_2_S level in plasma when compared with sham mice. NaHS injection could suppress the formation of atherosclerosis and degradation of both TGF-β and Smad3 phosphorylation, but PPG administration resulted in more decrease of TGF-β protein level and Smad3 phosphorylation and promote atherosclerosis formation. These findings preliminarily clarified the critical role of the antiatherosclerotic effect of CSE/H_2_S system via regulating TGF-β/Smad3 signaling pathway. This study is the first to report CSE/H_2_S system regulating TGF-β/Smad3 pathway in UAAS mice, suggesting that TGF-β/Smad3 signaling molecules is responsible for CSE/H_2_S system induced vascular protection against UAAS injuries of mice.

However, there were some limitations in this study. Firstly, we only evaluated the Scr and BUN levels which were widely used to assess renal function but didn’t show the histology of the remnant kidney. Secondly, we analyzed the lesion size of atherosclerotic plaque in the aortic roots, but didn’t quantify the effects of treatment on media calcification. Thirdly, the relationship between H_2_S and hypertension were not further explored in UAAS model.

## Conclusions

The CSE/H_2_S system is of great importance for treating atherosclerosis in patients with chronic kidney disease, and it may protect the vascular from atherosclerosis through the TGF-β/Smad pathway. The imbalance of CSE/H_2_S system may participate in the formation of UAAS.

## Supplementary Information

**Additional file 1: Figure S1.** Changes in Scr, BUN and levels of lipids in ApoE^−/−^ mice after 6 weeks operation. Scr and BUN and levels of lipids were increased in UAAS, UAAS+NaHS and UAAS+PPG group mice when compared with sham group mice.

**Additional file 2: Figure S2.** Effect of CSE/H_2_S system on aortic root lesion in UAAS mice at 2 to 8 weeks after operation. The aortic atherosclerotic plaque was widely observed in UAAS mice after 6 weeks operation. Compared with UAAS group, the aortic root lesions were significantly alleviated in UAAS+NaHS mice, but deteriorated in UAAS+PPG mice.

## Data Availability

The datasets used and/or analysed during the current study are available from the corresponding author on reasonable request.

## Abbreviations

H_2_S: Hydrogen sulfide
CSE: Cystathionine γ-lyase
UAAS: Uremia accelerated atherosclerosis
NaHS: Sodium hydrosulfide group
PPG: Propargylglycine
CKD: Chronic kidney disease
VCAM-1: Vascular cell adhesion molecule 1
ICAM-1: Intercellular adhesion molecule 1
CBS: Cystathionine β-synthase
3-MST: 3-mercaptopyruvate sulfurtransferase
Scr: Serum creatinine
BUN: Urea nitrogen
TC: Total cholesterol
TG: Triglyceride
LDL-C: Low-density lipoprotein-cholesterol
DTT: Dithiothreitol
SDS: Sodium dodecyl sulfate
PVDF: Polyvinylidene difluoride
DAB: Diaminobenzidine
