# Supplementary figures and images for: CSE/H2S system alleviates uremic accelerated atherosclerosis by regulating TGF-β/Smad3 pathway in 5/6 nephrectomy ApoE−/− mice
Source: BMC Nephrol. 2020 Dec 4;21:527. doi: 10.1186/s12882-020-02183-z (PMC7716493; doi:10.1186/s12882-020-02183-z)

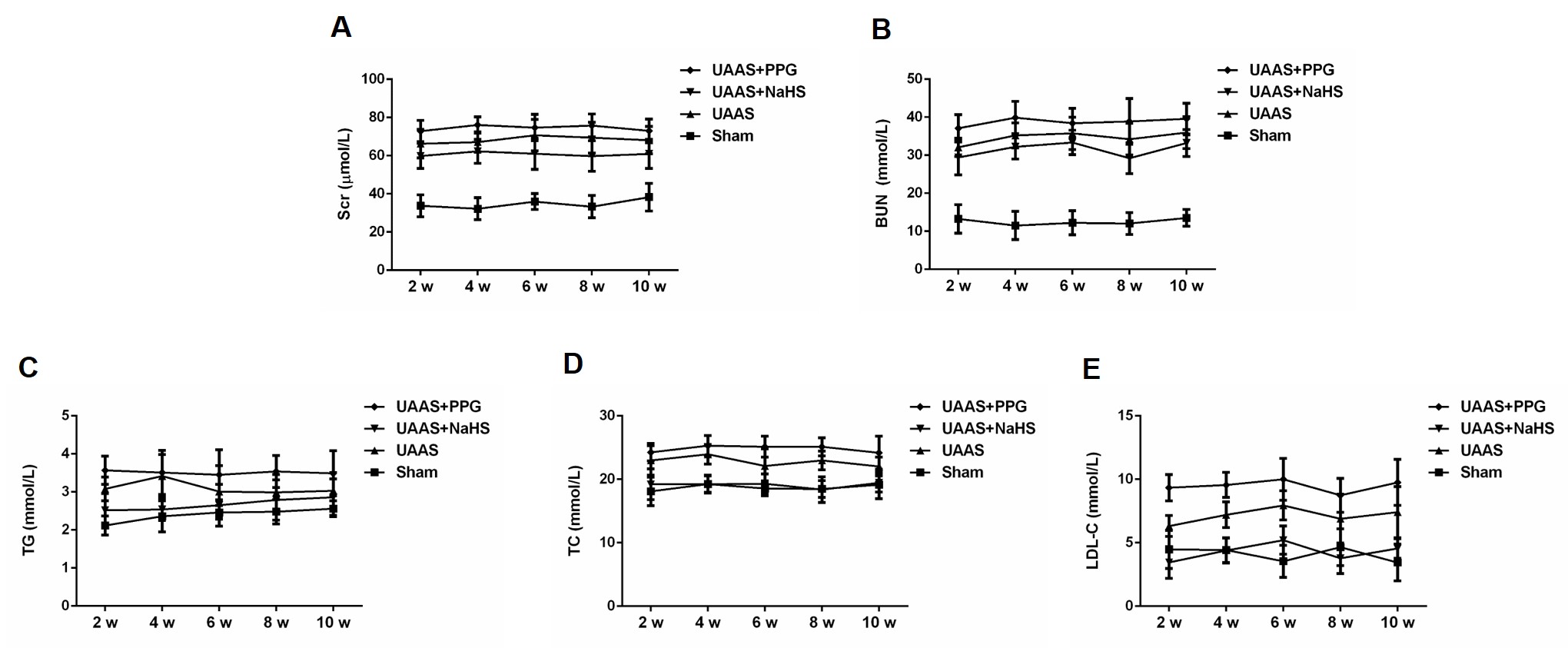

Supplement: Supplementary file 1 — Additional file 1: Figure S1. Changes in Scr, BUN and levels of lipids in ApoE−/− mice after 6 weeks operation. Scr and BUN and levels of lipids were increased in UAAS, UAAS+NaHS and UAAS+PPG group mice when compared with sham group mice. [file 12882_2020_2183_MOESM1_ESM.jpg]

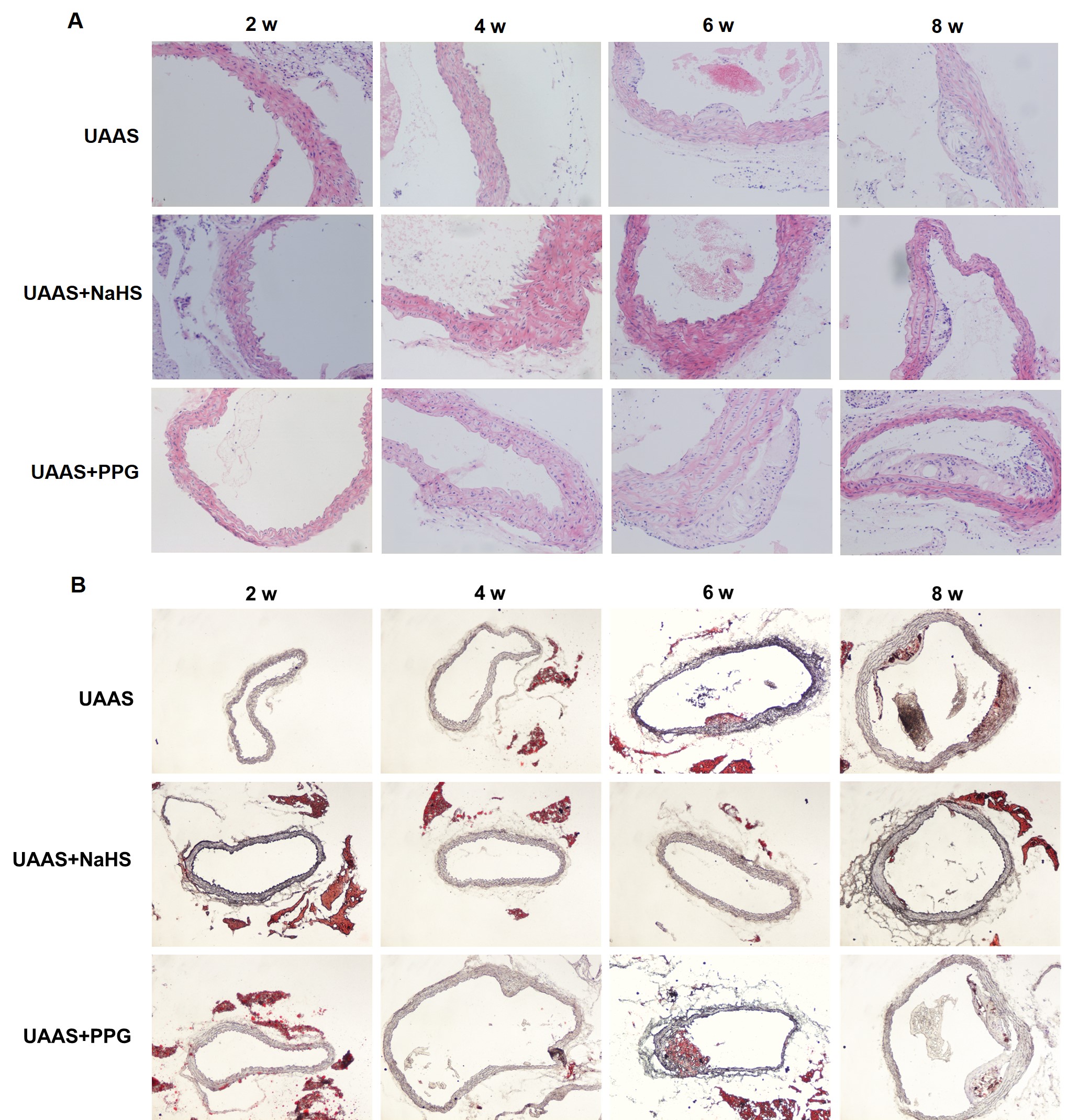

Supplement: Supplementary file 2 — Additional file 2: Figure S2. Effect of CSE/H2S system on aortic root lesion in UAAS mice at 2 to 8 weeks after operation. The aortic atherosclerotic plaque was widely observed in UAAS mice after 6 weeks operation. Compared with UAAS group, the aortic root lesions were significantly alleviated in UAAS+NaHS mice, but deteriorated in UAAS+PPG mice. [file 12882_2020_2183_MOESM2_ESM.jpg]
